# Utilizing apolipoprotein E genotypes and associated comorbidities for the assessment of the risk for dementia

**DOI:** 10.3389/fnagi.2022.927656

**Published:** 2022-12-12

**Authors:** Hsin Tung, Ching-Heng Lin, Yi-Ming Chen, Wei-Ju Lee, Li-Sheng Chien, Ting-Hsuan Sun, Cai-Sian Liao, Yung-Yang Lin, Tzu-Hung Hsiao

**Affiliations:** ^1^Institute of Clinical Medicine, National Yang Ming Chiao Tung University, Taipei, Taiwan; ^2^Department of Post-Baccalaureate Medicine, National Chung Hsing University, Taichung, Taiwan; ^3^Center of Faculty Development, Taichung Veterans General Hospital, Taichung, Taiwan; ^4^Neurological Institute, Taichung Veterans General Hospital, Taichung, Taiwan; ^5^Department of Medical Research, Taichung Veterans General Hospital, Taichung, Taiwan; ^6^Department of Public Health, College of Medicine, Fu Jen Catholic University, New Taipei, Taiwan; ^7^Institute of Public Health and Community Medicine Research Center, National Yang Ming Chiao Tung University, Taipei, Taiwan; ^8^Department of Industrial Engineering and Enterprise Information, Tunghai University, Taichung, Taiwan; ^9^Division of Allergy, Immunology and Rheumatology, Department of Internal Medicine, Taichung Veterans General Hospital, Taichung, Taiwan; ^10^Faculty of Medicine, School of Medicine, National Yang Ming Chiao Tung University, Taipei, Taiwan; ^11^Dementia Center, Taichung Veterans General Hospital, Taichung, Taiwan; ^12^Center for Geriatrics and Gerontology, Taichung Veterans General Hospital, Taichung, Taiwan; ^13^Institute of Brain Science, National Yang Ming Chiao Tung University, Taipei, Taiwan; ^14^Department of Critical Care Medicine, Taipei Veterans General Hospital, Taipei, Taiwan; ^15^Institute of Genomics and Bioinformatics, National Chung Hsing University, Taichung, Taiwan; ^16^Research Center for Biomedical Science and Engineering, National Tsing Hua University, Hsinchu, Taiwan; ^17^Department of Public Health, Fu Jen Catholic University, New Taipei City, Taiwan

**Keywords:** dementia, comorbidity, apolipoprotein E (*ApoE*), cerebrovascular accident (CVA), functional gastrointestinal disorder

## Abstract

**Introduction:**

Dementia is associated with many comorbidities while being related to Apolipoprotein E (*ApoE*) polymorphism. However, it is unclear how these clinical illnesses and genetic factors modify the dementia risk.

**Methods:**

We enrolled 600 dementia cases and 6000 matched non-dementia controls, with identified *ApoE* genotype (ε4/ε4, ε4/ε3, and ε3/ε3). Eight comorbidities were selected by medical records, and counted if occurring within 3 years of enrollment.

**Results:**

The dementia group had a higher ratio of carrying ε4 allele and prevalence of comorbidities than the non-dementia group. Homozygous ε4 carriers presented the broken line of dementia risk with the peak age at 65–75 years and odds ratio (OR) up to 6.6. The risk only emerged after 65 years of age in ε3/ε4 subjects with OR around 1.6–2.4 when aged > 75 years. Cerebrovascular accident (CVA) is the commonest comorbidity (14.6%). CVA, sleep disorder, and functional gastrointestinal disorders remained as significant risk comorbidities for dementia throughout all age groups (OR = 1.7–5.0). When functional gastrointestinal disorder and ε4 allele both occurred, the dementia risk exceeded the summation of individual risks (OR = 3.7 and 1.9 individually, OR = 6.0 for the combination). Comorbidities could also be predictors of dementia.

**Conclusion:**

Combining the genetic and clinical information, we detected cognitive decline and optimize interventions early when the patients present a specific illness in a particular age and carry a specific *ApoE* allele. Of comorbidities, functional gastrointestinal disorder is the strongest predicting factor for dementia in ε4 allele carriers.

## Introduction

Dementia is the most common neurodegenerative disease in the world, and the reported incidence and prevalence are around 8.6/1,000 person-years and 5.1%, respectively (Ponjoan et al., 2019). The classification of dementia depends on the neuropathological signs, but such features are usually overlapping and presenting as the spectrum (Raz et al., 2016). Demented patients have poor memory, impaired executive function, psycho-behavioral problems, and higher morbidity and mortality (Wolters and Ikram, 2018). Due to worldwide aging, the dementia population is gradually increasing. However, dementia remains incurable at present times. Only early intervention seems to delay dementia progression and prevent disability. Nevertheless, there is still a lack of standard guidelines in clinical practice on how to identify patients at risk of dementia early.

In addition to environmental and socioeconomic status (Jia et al., 2020), genetics is also an important risk factor for dementia. Apolipoprotein E (*ApoE*) polymorphism is the most susceptible genetic factor for late-onset Alzheimer’s disease (AD), which is located on chromosome 19, composed of 299 amino acids (Mahley and Rall, 2000). The amino acids at positions 112 and 158 determine three alleles (ε2, ε3, and ε4) (Mahley and Rall, 2000). The *ApoE* genotypes present differential effects on lipid-binding ability, amyloid-β (A-β) aggregation and clearance, and the susceptibility of the blood-brain barrier (Liu et al., 2013), and are linked to a neurodegenerative process (Mahley, 2016), including AD, vascular dementia, and mixed dementia (Liu et al., 2012). The heterozygous ε4 allele increased the AD risk by 2.6–3.2 times (Farrer et al., 1997; Houlden et al., 1998; Harold et al., 2009), while the homozygous ε4 allele further increased the risk by 14.9 times in Caucasians (Farrer et al., 1997). The ε4 allele effect seemed to possess ethnic differences, presenting 2.7–3.5 times (Lai et al., 2003) and 5 times (Zheng et al., 2016) higher dementia risk in the Chinese population, respectively. Besides, it is unclear how age modifies the risk related to *ApoE* polymorphism. Only one western cohort study demonstrated the reverse U-shaped dementia risk curve with the peak age at 70–75 years in both ε4 homozygotes and heterozygotes (Bonham et al., 2016). However, whether the Asian population has the same risk curve of *ApoE* polymorphism has not been disclosed.

Dementia subjects carry around two to eight chronic medical illnesses (Sanderson et al., 2002). Headache (Wang et al., 2018), ischemic stroke (Vijayan and Reddy, 2016), coronary artery disease (CAD) (Deckers et al., 2017), depression (Bennett and Thomas, 2014), sleep disturbance (Shi et al., 2018), fibromyalgia (Tzeng et al., 2018), and epilepsy (Sen et al., 2018) have been described as common comorbidities of dementia. In Taiwan’s cross-sectional study (Chen et al., 2017), diabetes mellitus (DM), cerebrovascular disease, liver cirrhosis, and asthma were found to be highly associated with cognitive decline. These chronic diseases seemed to have a bidirectional relationship with dementia because they might share similar risk factors, biological pathways, and environmental backgrounds. Besides, the *ApoE* genotype also affects the susceptibility to develop these illnesses (Afroze et al., 2016; Konialis et al., 2016; Burns et al., 2020). Oxidative stress, neuroinflammation, vascular risk factors, and blood-brain barrier breakdown are responsible for the development of both dementia and associated comorbidities (Raz et al., 2016). The relationship among *ApoE*, dementia, and comorbidities seems to be complex. How comorbidities interact with and influence cognitive function in the timeline has not been well-established. The role of these diseases, acting as a prodrome or as a predictor of dementia, remains unclear.

Therefore, we used a 3-year longitudinal case-control study to explore the interaction between *ApoE* polymorphism and clinical presentations. This is the first study that combined genetic and clinical information to identify the riskiest comorbidities to predict cognitive decline. Thus, the occurrence of such diseases in the subjects can be considered a red flag or a marker to inform clinicians to screen the cognitive function within a certain age population.

## Materials and methods

### Participants

The Taiwan Precision Medicine Initiative (TPMI) project began in June 2020. It has been promoted by Academia Sinica in Taiwan, and executed in 15 hospitals, including Taichung Veteran General Hospital (TCVGH). This study was approved by the ethics committee of Taichung Veterans General Hospital’s Institutional Review Board (CE16270B-1) in our hospital. We enrolled the outpatients who had the will to join this project and signed the informed consent. They received the genetic study using a customized SNP chip (Axiom Genome-Wide TWB 2.0 Array Plate) and provided their electrical medical records in our hospital. There were 46,020 participants enrolled from our hospital until the date we began the analysis in April 2021.

Because our main purpose is to study the risk of the ε4 allele of the *APOE* gene, we first identified the subjects carrying ε3/ε3, ε3/ε4, and ε4/ε4. There were 37,135 subjects which were enrolled among 46,020 participants. There were 600 newly diagnosed dementia cases between June 2013 and April 2021. Besides, 6,000 gender- and age-matched controls without dementia were selected, whose matched age was calculated by the year 2020.

### Apolipoprotein E genotyping

The DNA was extracted when the blood samples were obtained and then genotyped by the Genome-wide association study (GWAS) technique. We used the Axiom Genome-Wide TWB 2.0 Array Plate (Affymetrix, Santa Clara, CA, USA), which is designed for the Taiwan Han Chinese population. Affymetrix Power Tool was used for standard quality control. After excluding markers that failed Hardy–Weinberg equilibrium tests for controls (*P* < 1.0 × 10^–4^), minor allele frequency (*P* < 0.01), and the low call rate SNPs (<99%), there were 591,048 SNPs for analysis. This array contained two alleles of the *ApoE* gene: rs429358 and rs7412, which defined the three main *ApoE* alleles (ε2, ε3, and ε4). The ε3/ε3 carriers were used as a reference because it was the most common *ApoE* genotype.

### Disease identification

We included medical records from both inpatients and outpatients in the TCVGH from December 2009 to April 2021. The disease diagnosis was based on the International Classification of Disease-nine (ICD-9) code. All causes of dementia and cognitive decline disorders were collected (ICD-9 290.*, 294.0–294.1, 331.*). Because we set the development of dementia or cognitive decline as the endpoint, only the cases with their first diagnostic date between 1 January 2013 and 30 April 2021 were enrolled. The controls were selected from the subjects who were not diagnosed with dementia until 30 April 2021, who were age- (according to the year 2020) and gender-matched with dementia cases using the 1:10 ratio.

We selected eight common diseases presented as dementia-associated comorbidities and their first diagnosed date was recorded: headache (ICD-9 307.81, 346.*), epilepsy (ICD-9 345.*), cerebrovascular accident (CVA, ICD-9 433-438), cardiovascular disease (CAD, ICD-9 410-414), sleep disorders (ICD-9 307.4*, 780.5*), psychiatric disorders (ICD-9 290.8, 290.9, 294.8, 294.9, 295–297, 300.*, 311), functional gastrointestinal disorder (ICD-9 536.8, 536.9, 564.0, 564.1), and fibromyalgia (ICD-9 729.1). Besides, other vascular risk factors were also collected: diabetes (ICD-9 250.^**^) and hypertension (ICD-9 401.9). When dementia cases had any of these diseases diagnosed 3 months–3 years (90–1,095 days) before the diagnosis of dementia, it was defined as having this disease. In control groups, they were defined as having these diseases when the diseases occurred between 2018 and 2020. Because hypertension and diabetes are considered non-curable diseases, both were counted when they occurred before the diagnosis of dementia or the calculated year.

### Statistical analysis

SPSS version 20.0 (IBM, Armonk, NY, USA) was used for statistical analysis. Categorical variables were analyzed with a Chi-squared test for group comparison, while the student *t*-test was used for continuous variables. Clinical variables were analyzed by logistic regression. First, the presence of each comorbidity was applied to predict whether cognitive decline would occur after 3 months–3 years later. Then, the dementia risk was also calculated in three different age groups: younger than 65, 65–75, and older than 75 years. The statistical significance was defined as a *p*-value < 0.05.

## Results

### The dementia group had a higher prevalence of comorbidities

The baseline demographic characteristics and the presence of comorbidities in the two groups are shown in Table 1. *ApoE* genotypes are significantly different between the two groups. The dementia group showed a higher ratio of ε3/ε4 (23.8 vs. 16.2%) and ε4/ε4 (3.2 vs. 0.7%) than the control group. Within 3 years of dementia occurrence, the prevalence of most selected comorbidities and hypertension was higher in the dementia group than in the control group, except for a headache. Averagely, each dementia patient carried at least 0.6 comorbidities within 3 years before the diagnosis of dementia, which was 2.4 times of the age-comparable control group. The most common comorbidity was CVA, and it happened in at least one of the seven dementia cases (14.6%). Although CVA still showed the highest prevalence (5.8%) in the control group, it was only half of the value in the dementia group. For chronic diseases, diabetes presented a similar percentage in both groups (41.5% in the dementia group and 39.7% in the non-dementia group). The diagnostic ages of each disease are listed in Supplementary Table 1, which showed no significant differences between the demented and non-demented groups. In addition, the frequency of homozygous ε4 and ε3 cases were 0.63% (288/37,135) and 65.98% (30,363/37,135) in our hospital cohort, respectively (Supplementary Table 2).

**TABLE 1:** Demographic characteristics and associated diseases, which occurred 3 months–3 years before the diagnosis of dementia, in dementia, and matched non-dementia groups.

	Non-dementia (*n* = 6000)		Dementia (*n* = 600)		*P*-value*^$^*
	N	Percentage	N	Percentage	
Gender (M:F)		1:1.12		1:1.10	0.827
Male	2,832	47.2%	286	47.7%	
Female	3,168	52.8%	314	52.3%	
Age	73.0	±9.3	73.0	±9.4	0.942^#^
**Genotype**					
ε3/ε3	4,984	83.1%	438	73.0%	**<0.001**
ε3/ε4	974	16.2%	143	23.8%	**<0.001**
ε4/ε4	42	0.7%	19	3.2%	<0**.001**
**Comorbidities:**					
Headache	75	1.3%	10	0.2%	0.388
Epilepsy	29	0.5%	8	1.3%	**0.008**
CVA	345	5.8%	88	14.6%	**<0.001**
CAD	338	5.6%	54	9.0%	**0.001**
Sleep disorder	168	2.8%	62	10.3%	**<0.001**
Psychiatric disorder	200	3.3%	34	5.6%	**0.003**
Functional GI disorder	214	3.6%	68	11.3%	**<0.001**
Fibromyalgia	156	2.6%	49	8.2%	**<0.001**
Hypertension	2,714	45.2%	301	50.2%	**0.021**
Diabetes	2,384	39.7%	249	41.5%	0.400

^$^chi-squared test.

#Mann-Whitney U test.

CVA, cerebrovascular accident; CAD, coronary artery disease; GI, gastrointestinal; SD, standard deviation; M, male; F, female.

### Dementia was associated with the ε4 allele and some comorbidities

In the univariate analysis, almost all selected comorbidities were associated with dementia occurrence within 3 months–3 years later, except for headache and diabetes (Table 2). By applying multivariate regression, the odds ratio (OR) of cognitive decline for carrying any *ApoE* ε4 allele was 1.89 in contrast to the ε3/ε3 polymorphism. In the clinical aspect, we found that CVA, CAD, sleep disorders, functional gastrointestinal disorders, and fibromyalgia had a significantly higher OR for the occurrence of dementia within 3 months–3 years, ranging from 1.43 to 3.09. Among them, sleep disorders were the most dangerous factor, presenting 3.09 OR for dementia occurrence, followed by functional gastrointestinal disorders with OR of 2.73. Neither these two common chronic diseases, hypertension and diabetes, showed a higher risk for dementia after multivariate regression.

**TABLE 2:** Use of logistic regression to predict dementia development between 3 months and 3 years of presence of comorbidities.

	Univariate			Multivariate		
	Odds ratio	95% CI	*P*-value^#^	Odds ratio	95% CI	*P*-value^#^
Age (years)	1.00	(0.99–1.01)	0.975	1.00	(0.99–1.01)	0.842
APOE genotype (any e4 allele)						
ε 4/ε 4 &ε 4/ε 3 vs. ε 3/ε 3	1.81	(1.50–2.20)	<0.001	**1.89**	**(1.55**–**2.31)**	**<0.001**
Headache	1.34	(0.69–2.60)	0.390	0.82	(0.40–1.69)	0.587
Epilepsy	2.78	(1.27–6.11)	**0.011**	1.82	(0.80–4.17)	0.155
CVA	2.82	(2.19–3.62)	**<0.001**	2.22	(1.69–2.90)	**<0.001**
CAD	1.66	(1.23–2.24)	**0.001**	1.43	(1.05–1.96)	**0.023**
Sleep disorder	4.00	(2.95–5.42)	**<0.001**	**3.09**	**(2.23**–**4.28)**	**<0.001**
Psychiatric disorders	1.74	(1.20–2.53)	**0.004**	1.22	(0.82–1.82)	0.334
Functional GI disorder	3.46	(2.59–4.60)	**<0.001**	**2.73**	**(2.01**–**3.71)**	**<0.001**
Fibromyalgia	3.33	(2.39–4.65)	**<0.001**	2.12	(1.48–3.04)	**<0.001**
Hypertension	1.22	(1.22–1.44)	**0.021**	1.12	(0.93–1.35)	0.216
Diabetes	1.08	(0.91–1.28)	0.400	1.04	(0.86–1.24)	0.715

^#^Logistic regression.

CVA, cerebrovascular disease; CAD, coronary artery disease; GI, gastrointestinal. Bold values indicated that this item presented statistical significance.

### Dementia risk related to comorbidities and the ε4 allele varies with age

After age stratification, homozygous *ApoE* ε4 carriers presented the highest risk among all factors for dementia occurrence throughout all age groups (Table 3). Its OR reached the peak in the group of 65–75 years up to 6.63, and then, the risk declined after 75 years to 4.06. Heterozygous ε4 carriers showed dementia risk only when they were older than 65 years. The OR gradually increased with the age, from 1.61 to 2.40 at the age older than 65 years. Among comorbidities, CVA, sleep disorders, and functional gastrointestinal disorders remained significant risk factors for dementia occurrence within 3 years throughout all age groups, and the OR ranged from 1.74 to 5.05. Both CVA and sleep disorders showed the highest risk in the relatively younger age group (<65 years) than in the older group (>65 years). Functional gastrointestinal disorder showed the highest risk within the 65–75 years group. Besides, the presence of fibromyalgia only demonstrated dementia risk in the age of 65–75 years. CVA (OR 5.05), functional gastrointestinal dysfunction (OR 3.04), and sleep disorders (OR 3.29) were the most dangerous comorbidities in the younger than 65, 65–75, and older than 75 years age groups, respectively.

**TABLE 3:** Multivariate logistic regression predicts dementia development between 3 months and 3 years of presence of comorbidities in different age groups.

	<65 year-old (*n* = 1,122) [Dementia = 102, 9.1%]			65–75 year-old (*n* = 2,795) [Dementia = 225, 8.1%]			>75 year-old (*n* = 2,683) [Dementia = 243, 9.1%]		
	Odds ratio	95% CI	*P*-value^#^	Odds ratio	95% CI	*P*-value^#^	Odds ratio	95% CI	*P*-value^#^
APOE genotype			**0.002**			**<0.001**			**<0.001**
ε 4/ε 3 vs.ε 3/ε 3	0.73	(0.38–1.40)	0.345	**1.61**	**(1.18**–**2.22)**	**0.003**	**2.40**	**(1.76**–**3.254)**	**<0.001**
ε 4/ε 4 vs.ε 3/ε 3	**6.53**	**(2.20**–**19.36)**	**0.001**	**6.63**	**(3.09**–**14.22)**	**<0.001**	**4.06**	**(1.06**–**15.48)**	**0.040**
Headache	0.43	(0.07–2.58)	0.357	0.71	(0.22–2.28)	0.561	1.23	(0.41–3.72)	0.717
Epilepsy	4.35	(0.64–29.44)	0.132	2.59	(0.86–7.78)	0.090	0.67	(0.08–5.44)	0.711
CVA	**5.05**	**(2.60**–**9.81)**	**<0.001**	**1.74**	**(1.11**–**2.72)**	**0.016**	**2.18**	**(1.45**–**3.27)**	**<0.001**
CAD	1.26	(0.56–2.85)	0.574	1.56	(0.98–2.50)	0.061	1.40	(0.85–2.30)	0.193
Sleep disorder	**4.32**	**(1.99**–**9.37)**	**<0.001**	**2.56**	**(1.54**–**4.26)**	**<0.001**	**3.29**	**(1.93**–**5.59)**	**<0.001**
Psychiatric disorders	2.58	(0.99–6.75)	0.053	1.01	(0.52–1.98)	0.977	1.16	(0.63–2.12)	0.638
Functional GI disorders	**2.43**	**(1.06**–**5.54)**	**0.035**	**3.04**	**(1.91**–**4.84)**	**<0.001**	**2.51**	**(1.53**–**4.12)**	**<0.001**
Fibromyalgia	2.21	(0.88–5.57)	0.092	**2.55**	**(1.50**–**4.34)**	**0.001**	1.72	(0.93–3.20)	0.085
Hypertension	0.74	(0.44–1.24)	0.253	1.07	(0.81–1.41)	0.651	1.29	(0.96–1.72)	0.093
Diabetes	1.33	(0.82–2.15)	0.253	1.05	(0.79–1.40)	0.721	0.97	(0.73–1.30)	0.849

^#^Logistic regression.

CVA, cerebrovascular disease; CAD, coronary artery disease; GI, gastrointestinal. Bold values indicated that this item presented statistical significance.

### Functional gastrointestinal disorders have a further higher risk for dementia in ε4 allele carriers

**FIGURE 1:**
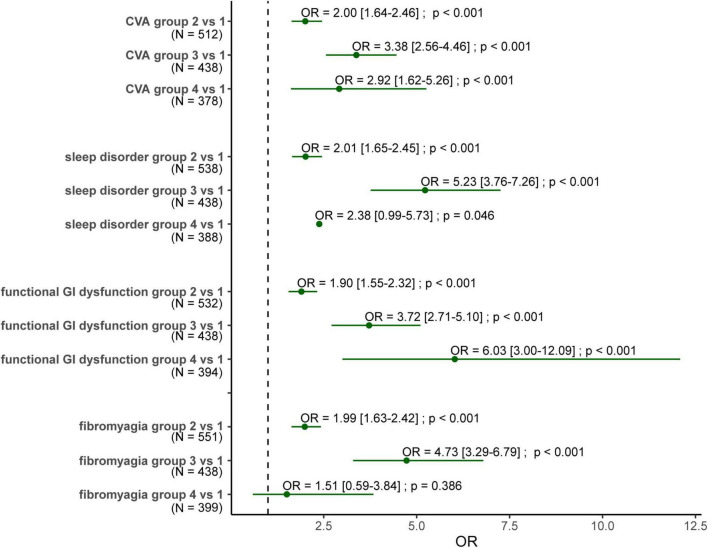
Forest plot for the odds ratio of dementia for subjects carrying ε4 allele with/without comorbidities. Group 1: subjects without the ε4 allele nor the comorbidity. Group 2: subjects carrying the ε4 allele without the comorbidity. Group 3: subjects carrying the comorbidity without the ε4 allele. Group 4: subjects carrying both the ε4 allele and the comorbidity. OR, odds ratio; N, the number of the demented cased of these two groups. The numbers within the square brackets are confidence interval.

We took the four most important risk factors (ε4 allele, CVA, sleep, and functional gastrointestinal disorders), which were selected by multivariate analysis from Table 3, to explore the combined effect of genes and comorbidities on dementia (Figure 1). The ε4 allele carriers had a persistently higher risk for dementia compared with those not carrying ε4, with OR around 1.9–2.0. These three comorbidities alone were also related to the risk of dementia, and OR ranged from 3.4 to 5.3. When combining the ε4 allele and the comorbidities, these three comorbidities presented different patterns of dementia risk. Subjects having CVA with or without the ε4 allele show a similar risk of dementia (OR 2.9 vs. 3.4). When functional gastrointestinal disorders and the ε4 allele both occurred, the risk of dementia exceeded the summation of individual risks (OR 3.7 and 1.9 individually, OR 6.0 for the combination), demonstrating a further higher dementia risk. In contrast, the presence of sleep disorders alone showed the highest risk (OR 5.2) compared to the ε4 allele with and without sleep disorders (OR 2.4 and 2.0).

## Discussion

This hospital-based case-control study demonstrated the impact of specific diseases/events and the *ApoE* polymorphism on dementia occurrence. This is the first study to explore how clinical and genetic factors affect dementia risk individually and collectively. We combined the ε4 allele and comorbidities as factors to establish a model to predict the occurrence of dementia within 3 years. Therefore, it allows us to screen for dementia and provide early intervention once these risky comorbidities occur in ε4 carriers.

Among the studied risk factors, the ε4 allele of the *ApoE* genotype is the persistently significant factor to predict dementia throughout all age groups. Our ε4 allele carriers had around two times higher risk for all-cause dementia within 3 years compared with ε3/ε3 carriers, which seemed to be relatively lower compared with previous case-control studies. The OR for subjects carrying at least one ε4 allele was 3.84 for late-onset AD and 1.70 for vascular dementia in Han Chinese (Yang et al., 2001). In Caucasians, the odds ratio for AD was 3.2 in ε3/ε4 and 14.9 in ε4/ε4 compared with the ε3/ε3 allele (Farrer et al., 1997). In contrast to such studies, our non-demented controls were selected, enrolling age- and gender-matched subjects, instead of only non-demented cases. This method avoided the confounding effect of age since the dementia group might be older.

After age stratification, the risk of dementia in ε3/ε4 carriers only emerged after 65 years. However, the ε4/ε4 carriers had a persistently higher dementia risk in all age groups. This phenomenon suggested the additive effect of the ε4 allele, for which the number of ε4 alleles is correlated with the dementia risk and the onset age (Liu et al., 2013). Although *ApoE* polymorphism had been told to show extensive influence from the age of 40–90 years (Farrer et al., 1997), the strength of the risk seemed to change with age. Instead of a reverse U-shaped risk curve for dementia observed in the western literature (Bonham et al., 2016), our heterozygous and homozygous ε4 carriers demonstrated separate trends of the age-dependent risk. The ε3/ε4 carriers presented a slowly upgoing line. In contrast, we found that the dementia risk seemed to reach the peak at the age of 65–75 years in ε4/ε4 carriers, and then the genetic effect declined after 75 years. However, because only 19 dementia cases were diagnosed in our ε4/ε4 carriers, we could not confirm whether the presence of the broken line in homozygous ε4 subjects was due to a smaller sample size. The ε3/ε4 and the ε4/ε4 had different routes for dementia occurrence in Han Chinese. The strategy of risk assessment for dementia screening could not be the same for these two groups. The ε4 homozygotes need more active surveillance and earlier prevention.

The dementia subjects were reported to carry around 2.4 comorbidities (Schubert et al., 2006), and we found 0.6 comorbidities occurred within 3 years of dementia being diagnosed. Among the listed comorbidities, CVA, sleep disorders, and functional gastrointestinal disorders seemed to be the important risk factors to predict dementia occurrence within 3 years.

Occurrence of CVA or stroke was found to have 1.7–5.0 times of risk for cognitive decline. Stroke has been considered the major risk factor for vascular dementia and AD for a long time (Vijayan and Reddy, 2016). The risk of post-ischemic stroke dementia was 1.7–3.8 (Desmond et al., 2002), and the risk was much higher in ε4 homozygotes (Pendlebury et al., 2020). The incidence of new-onset dementia gradually increased after the index stroke, reporting from 7% in the first year to 10% in the third year, and 23% in the tenth year (Zhu et al., 2000). Stroke leads to intracranial hypoxia, provoking oxidative stress (Slevin et al., 2015), inflammation, and microRNA alteration, which then facilitate and accelerate A-β protein accumulation (Vijayan and Reddy, 2016), and finally, dementia pathogenesis (Brenowitz et al., 2021). We found that the post-stroke dementia risk is highest in the relatively younger group, which appeared in the reverse direction of the stroke incidence and prevalence that increases with age (Sedova et al., 2021). We postulated that the younger brain is more susceptible to the ischemic insult, which might be related to a more fulminant inflammatory response. *ApoE* ε4 also disturbs lipid homeostasis in astrocytes and microglia and then leads to blood-brain barrier failure in stroke patients (Duong et al., 2021). Furthermore, the ε4 allele is also related to a younger stroke age (Lagging et al., 2019) and a higher ischemic stroke risk (Konialis et al., 2016). We found that the ε4 allele and stroke would individually increase the dementia risk, but this risk was not summative when both factors were simultaneously present. Therefore, when subjects had the first event of a stroke, especially at a younger age, it is necessary to keep an eye on the subtle signs of dementia occurrence.

Sleep disturbance showed a 2.5–4.3-times higher risk for future cognitive decline while it was reported to increase the risk of dementia by 50–80% in previous studies (Shi et al., 2018; Brenowitz et al., 2021). On the molecular level, abnormal sleep duration is associated with amyloid-β accumulation (Winer et al., 2021) and sleep-disordered breathing increased tau deposition (Bubu et al., 2019). The dementia risk was also relatively higher in subjects aged less than 65 years in our study, reflecting that the younger brain is vulnerable to such abnormal protein accumulation. However, there was no evidence that *ApoE* genotypes have differential effects on sleep disorders (Palpatzis et al., 2021). This phenomenon explained our finding in Figure 1 that subjects who had only sleep disorders presented with a significantly higher dementia risk regardless of carrying the ε4 allele. Therefore, sleep disorder itself seems to jeopardize dementia risk more than the *ApoE* genotype does. Thus, sleep disturbance might be a potentially modifiable risk factor to prevent dementia, even if there has been insufficient evidence to confirm it yet.

Gastrointestinal dysfunctions, such as constipation and delayed gastric emptying, are associated with a 2.4–3.0-times higher dementia risk throughout all age groups in our study. Gut microbiota alteration occurred several years before the appearance of cognitive decline (Li et al., 2019), which indicated that the subjects had been exposed to systemic disturbances related to metabolites of the gut microbiota, such as lipopolysaccharide and trimethylamine. They influence the neurological system by affecting neurotransmitters (Zhuang et al., 2020), such as serotonin and GABA, and increasing amyloid-β deposition (Nagpal et al., 2019). Consequently, the theory of the gut-brain axis had been proposed in many neurodegenerative disorders (Martin et al., 2018). However, whether probiotic treatment will prevent dementia development after a change in the intestinal ecology is still unknown (Santiago and Potashkin, 2021). Besides, *ApoE* polymorphism is also associated with the compositions of the gut microbiome (Parikh et al., 2020). This phenomenon is also reflected in our observation. When ε4 allele carriers presented any functional gastrointestinal disorder, their risk for developing dementia within 3 years further increased, exceeding those having any one of the two factors. Among comorbidities, functional gastrointestinal disorders might act as an important red flag sign in ε4 allele carriers. Accordingly, even though gastrointestinal symptoms are usually non-specific, they might be used as a biomarker for early detection and prediction of cognitive decline in ε4 allele carriers in clinical practice.

Cerebrovascular accident, sleep disorders, functional gastrointestinal dysfunctions, and fibromyalgia seem to be the predictors of dementia with variable risk based on age, suggesting the susceptibility of the brain changes with age. Although we could not confirm the cause-effect relationship between dementia development and comorbidity occurrence, the interaction of *ApoE* polymorphism and these clinical illnesses certainly modifies the dementia risk. Combining the genetic and clinical information, we have the odds to early detect cognitive decline and optimize interventions when the subjects first present with a specific illness at a particular age and carry a specific *ApoE* allele.

There were some limitations in our study. First, the diagnosis of dementia and associated diseases was based on the medical records only from our hospital. We could miss the diagnoses which had been made in other hospitals. Second, the illness with a minor severity, such as headaches or gastrointestinal discomforts, could also be ignored if the subjects did not search for medical assistance. Underestimation of the risk related to these illnesses might be possible. Besides, instead of further classifying dementia subtypes, such as vascular dementia and AD, we took all-cause cognitive decline as the single group for analysis. Furthermore, our sample size was not large enough because only 600 dementia cases were identified. It made the statistical model unstable. We had the preservation to conclude the interaction between the *ApoE* genotype and comorbidities, as well as the dementia risk related to individual comorbidities in three age groups.

## Conclusion

Our study demonstrated that dementia patients not only had a higher chance to carry the ε4 allele, but also had a higher prevalence of many chronic illnesses within 3 years before the cognitive decline, including hypertension, headache, epilepsy, CVA, CAD, sleep disorders, psychiatric disorders, functional gastrointestinal disorders, and fibromyalgia. Although *ApoE* polymorphism seems to be the riskiest factor for dementia, the effects of the number of ε4 alleles differ. The ε4 homozygotes presented a persistently high risk for dementia in all age groups, while the risk only emerged after 65 years in ε3/ε4 subjects. Among these comorbidities, the occurrence of CVA, sleep disorders, functional gastrointestinal disorders, and fibromyalgia seemed to be predictive of cognitive decline within 3 years. Besides, functional gastrointestinal disorders might be an important predicting factor for dementia occurrence in ε4 allele carriers.

## Data availability statement

The clinical data presented in this study are available on request from the corresponding authors. The genetic data from the Taiwan Precision Medicine Initiative are not publicly available because some access restrictions may apply to the data underlying the findings. The data used in this study cannot be made available in the manuscript, the supplemental files, or in a public repository due to the Personal Information Protection Act executed by Taiwan’s government, starting in 2012. Requests for data can be sent as a formal proposal to obtain approval from the ethics review committee of the appropriate governmental department in Taiwan.

## Ethics statement

The studies involving human participants were reviewed and approved by the Institutional Review Board of Taichung Veterans General Hospital (TCVGH) (CE20316A). The patients/participants provided their written informed consent to participate in this study.

## Author contributions

HT, C-HL, Y-MC, and T-HH: work conception and study design. HT and W-JL: data acquisition and collection. T-HS and C-SL: conducting NGS and genomic data analysis. HT and L-SC: clinical data analysis. C-HL and Y-MC: interpretation of data. HT and T-HH: drafting the work. Y-YL and T-HH: revising the work for valuable intellectual content. HT, Y-YL, and T-HH: final approval of the version. All authors contributed to the article and approved the submitted version.
