# Supplementary material for: Utilizing apolipoprotein E genotypes and associated comorbidities for the assessment of the risk for dementia
Source: Front Aging Neurosci. 2022 Dec 12;14:927656. doi: 10.3389/fnagi.2022.927656 (PMC9790994; doi:10.3389/fnagi.2022.927656)
Supplement: Supplementary file 1 [file Data_Sheet_1.docx]

Supplementary Material

**Supplementary Table 1. Demographic characteristics and associated diseases, which occurred 3 months to 3 years before the diagnosis of dementia, in dementia and matched non-dementia groups**

|  | **Non-dementia (n=6000)** | | | | | **Dementia (n=600)** | | | |  |  | |
| --- | --- | --- | --- | --- | --- | --- | --- | --- | --- | --- | --- | --- |
|  | **case number** | **ratio** | **mean age** | **SD** | **case number** | | **ratio** | **mean age** | **SD** | ***p value^#^ (age)*** | |  |
| **Gender (M:F)** | 2832:3168 | 1:1.12 |  |  | 286:314 | | 1:1.10 |  |  |  | |  |
| **Age** |  |  | 73.0 | +/- 9.3 |  | |  | 73.0 | +/- 9.4 | 0.942 | |  |
| **Genotype** | |  |  |  |  | |  |  |  |  | |  |
| **ε3/ε3** | 4984 | 83.1% | 73.2 | +/-9.3 | 438 | | 73.0% | 72.5 | +/- 9.6 | 0.438 | |  |
| **ε3/ε4** | 974 | 16.2% | 72.6 | +/- 9.3 | 143 | | 23.8% | 75.2 | +/- 8.1 | 0.054 | |  |
| **ε4/ε4** | 42 | 0.7% | 69.3 | +/- 7.4 | 19 | | 3.2% | 68.4 | +/- 9.6 | 0.341 | |  |
| **Comorbidities:** |  |  |  |  |  | |  |  |  |  | |  |
| **Headache** | 75 | 1.3% | 70.9 | +/- 9.7 | 10 | | 0.2% | 70.9 | +/- 7.7 | 0.467 | |  |
| **Epilepsy** | 29 | 0.5% | 73.2 | +/- 8.9 | 8 | | 1.3% | 67.9 | +/- 8.1 | 0.605 | |  |
| **CVA** | 345 | 5.8% | 73.3 | +/- 8.7 | 88 | | 14.6% | 70.8 | +/- 9.5 | 0.146 | |  |
| **CAD** | 338 | 5.6% | 71.1 | +/- 9.2 | 54 | | 9.0% | 71.6 | +/- 8.4 | 0.415 | |  |
| **Sleep disorder** | 168 | 2.8% | 71.2 | +/- 8.6 | 62 | | 10.3% | 70.2 | +/- 9.1 | 0.131 | |  |
| **Psychiatric disorder** | 200 | 3.3% | 74.2 | +/- 9.0 | 34 | | 5.6% | 70.1 | +/- 9.9 | 0.606 | |  |
| **Functional GI disorder** | 214 | 3.6% | 71.8 | +/- 8.7 | 68 | | 11.3% | 70.3 | +/- 9.1 | 0.425 | |  |
| **Fibromyalgia** | 156 | 2.6% | 71.5 | +/- 9.3 | 49 | | 8.2% | 69.6 | +/- 8.6 | 0.946 | |  |
| **Hypertension** | 2714 | 45.2% | 68.6 | +/- 9.2 | 301 | | 50.2% | 70.4 | +/- 8.7 | 0.303 | |  |
| **Diabetes** | 2384 | 39.7% | 68.2 | +/- 9.3 | 249 | | 41.5% | 68.8 | +/- 8.6 | 0.496 | |  |

SD = standard deviation

M=male, F=female

#: student T test

CVA= cerebrovascular accident, CAD = coronary artery disease, GI = gastrointestinal

**Supplementary Table 2. Demographic characteristics and distribution of three APOE genotypes.**

|  | **ε4/ε4** | **ε3/ε4** | **ε3/ε3** |
| --- | --- | --- | --- |
| **Case number**  **(totally: 37135)** | **288 (0.63%)** | **6484 (14.09%)** | **30363 (65.98%)** |
| **Gender (M:F)** | (1:1.09) | (1:1.19) | (1:1.19) |
| **Male** | 138 (47.9%) | 2963 (45.7%) | 13876 (45.7%) |
| **Female** | 150 (52.1%) | 3521 (54.3%) | 16487 (54.3%) |
| **Age (in 2021)** |  |  |  |
| **mean +/- SD** | 57.4 +/- 14.3 | 58.1 +/- 14.8 | 58.1 +/- 15.2 |
